# Domains of Well-Being in Minimally Conscious Patients: Illuminating a Persistent Problem

**DOI:** 10.1080/21507740.2018.1460415

**Published:** 2018-06-05

**Authors:** Mackenzie Graham

**Affiliations:** University of Oxford

Strahl and Banja (2018) bring needed attention to the challenges of balancing the precedent autonomy and quality of life concerns of patients in the minimally conscious state (MCS). MCS patients possess severely altered consciousness, but are capable of demonstrating inconsistent behavioral evidence of awareness of self, or environment. Strahl and Banja argue that appealing to precedent autonomy is not a panacea for surrogate decision making on behalf of MCS patients. For this reason, we need to appeal to considerations of patient quality of life to supplement decisions to maintain or withdraw life-sustaining treatment.

Assessing the quality of life of patients who are only intermittently conscious, are largely noncommunicative, and retain unknown cognitive capacity is just as difficult as it sounds. On the one hand, we have limited knowledge of the subjective experiences of MCS patients. While there is neurophysiological evidence to suggest that MCS patients can feel pain (Boly et al. 2008), for example, it remains unclear what else they experience. On the other hand, suppose MCS patients are capable of experiencing a variety of mental states, beyond physical pleasure and pain. How do these experiences contribute to or detract from their quality of life? Individuals often undergo a significant shift in their desires, attitudes, goals, and values after a major injury or disease, which makes it difficult to ascertain what a patient’s current values or interests might be. The barriers to communication with MCS patients itself poses a major hurdle to investigating their quality of life directly.

An alternative strategy is to consider the subjective experiences of other patient populations, outside those with disorders of consciousness. While MCS patients are a unique population, there may be some commonality between the quality of life concerns of MCS patients and other patients with severe physical and/or cognitive limitations. Examining the domains of life that these other patients consider central to their quality of life could provide some insight into the domains of life relevant to MCS patients, and provide a starting point for further inquiry.

First, consider individuals with a common etiology: Severe traumatic brain injury. MCS patients are one subset of patients with traumatic brain injury, and may share a similar collection of cognitive and behavioral impairments including deficits in attention, memory, and executive function, as well as anxiety, depression, and motor dysfunction (Carlozzi, Tulsky, and Kisala 2011). Research assessing the quality of life of individuals with traumatic brain injury has demonstrated that social participation, specifically interpersonal relationships, independence, and autonomy, is a centrally important domain of quality of life for these patients. Physical and medical health has also been highlighted (especially motor function), as well as cognitive health and personality change (Carlozzi, Tulsky, and Kisala 2011; Dijkers 2004).

A second relevant patient population is patients with severe motor impairments, specifically, patients with locked-in syndrome and patients with amyotrophic lateral sclerosis (ALS). Locked-in patients are incapable of voluntary movement (except, in most cases, for vertical eye movement) or verbal communication, although they remain fully aware. Similarly, ALS causes the death of motor neurons that control voluntary muscles, resulting in motor dysfunction and eventual paralysis, as well as an inability to swallow or breathe without mechanical assistance. (Like locked-in patients, ALS patients retain relatively normal levels of cognitive function.) Several studies have found that the self-reported quality of life of locked-in patients is within the same general range as that of healthy individuals (Bruno et al. 2011; Laureys et al. 2005). One research study found that 72% of locked-in syndrome patients were happy overall, with 82% of respondents satisfied with their personal relationships with others. They also reported that only 21% of respondents were engaged most of the day in activities they considered “important,” while 12% of respondents were dissatisfied with their participation in recreational activities, and 40% dissatisfied with their social participation (Bruno et al. 2011). Another study found that the ability to communicate with others and the ability to participate in family and community life are significant determinants of quality of life in patients with locked-in syndrome (Rousseau et al. 2013). Similarly, studies of patients with ALS suggest that psychological, supportive, and spiritual factors are most associated with patient welfare, particularly the patient’s perception of the quality of social and family support (Neudert, Wasner, and Borasio 2001; Simmons et al. 2006).

A third comparison population for MCS patients is patients with late-stage Huntington’s disease. Huntington’s disease is a fatal neurodegenerative disease resulting in severe motor, cognitive, and psychiatric disturbances, over a period of 15–20 years after symptom onset. Research involving these patients has found several specific domains relevant to overall quality of life: cognitive factors (e.g., memory, concentration, comprehension), psychology (e.g., personal/family worries about disease), adequate services, physical and functional factors (e.g., motor impairment), mood, and self and vitality (e.g., motivation, social role, feelings of isolation) (Clay et al. 2012; Hocaoglu, Gaffan, and Ho 2012).

By examining the factors contributing to the well-being of these comparison populations, I suggest that there are several broad themes that are applicable to MCS patients. First, there is good evidence to suggest that even in the face of severe mental or physical disability (or both), patients may report a reasonably high quality of life, often much higher than we would expect based on the severity of their condition. Indeed, there is the potential for underestimating the quality of life of these patients, especially when we attempt to determine their quality of life in terms of those values that seem to be most relevant to healthy individuals. Because there is the possibility for a change in patient values, we must exercise caution in simply assuming that the welfare of these patients is poor, without first reflecting on the basis upon which we are making this evaluation, and whether or not this is how the patient would evaluate their lives.

Second, the preceding examples suggest that when an individual has a severe chronic illness or disability that limits his or her physical, mental, or social function in some way, those domains of life that are impaired eventually come to be viewed as less important for quality of life. Patients do seem to adapt to their conditions. This suggests that MCS patients may adapt to their circumstances as well, such that the domains of life that remain accessible to them in some way may come to be viewed as more central to their welfare than those that are no longer accessible. For example, positive hedonic experiences—including physical pleasure and the absence of physical pain or discomfort, but also the pleasure of listening to music or an audiobook, being in the presence of a loved one, watching an enjoyable movie, or being out in nature—are likely to be things that contribute to their welfare. Further research into the residual cognitive capacities of patients with disorders of consciousness, including MCS patients, is needed, because it will provide insight into the kinds of experiences that might contribute to their quality of life. Rather than focusing on the ways in which their condition has disrupted their life plans, or previously held desires, it seems possible that MCS patients might focus on those aspects of life from which they can still benefit.

Third, based on the patient populations examined, there appears to be at least a general agreement that relationships with others and perceived social support are two domains that are particularly important for quality of life among such patients. Interestingly, many of these patients consider their physical or cognitive limitations to be less relevant to their quality of life, while emotional and social domains are seen as having a greater relevance.

The importance of emotional and social domains for quality of life highlights the need for attending to these domains in the care of MCS patients. Treatment and care efforts should focus not only on physical aspects of quality of life, but on emotional aspects as well. Hospital staff members should acknowledge the potential awareness of MCS patients in their interactions with them. Talking to the patients, guiding them through care procedures, identifying them by their own names, and avoiding parallel conversations recognizes that they are still persons. Insofar as these patients are capable of emotional experiences or relationships with others, attending to these domains may be highly important to their subjective welfare.

Summarily, quality-of-life research in the patient populations discussed here underscores the need for further neuroscientific research investigating the subjective experiences of MCS patients. MCS patients may retain a range of cognitive capacities, and the extent of their cognitive capacities may influence those domains that are most relevant to their quality of life. At the same time, consideration of comparison populations suggests that we can influence the quality of life of MCS patients in positive ways. While it may be difficult to improve a patient’s level of physical or cognitive function, attention to a patient’s emotional and social needs may provide a practical avenue for improving their welfare.
